# PROXIMAL DETERMINANTS OF FALLS IN OLDER ADULTS: THE MOBILIZE BOSTON STUDY

**DOI:** 10.1093/geroni/igz038.1749

**Published:** 2019-11-08

**Authors:** Hyun Gu Kang, Jonathan Hsu

**Affiliations:** California State University San Marcos, San Marcos, California, United States

## Abstract

Objectives: To study risk factors for falling, we examined risk factors for falls among older people according to the proximal determinants present at the time of the fall. Methods: Data came from MOBILIZE Boston, a prospective cohort study of 765 community-dwelling women and men, mainly aged 70 years or older. Over 4.3 years, 1737 falls were recorded, along with narrative reports describing proximal determinants at the time of the fall. Proximal determinants were identified from narrative reports and falls were classified into categories. Categorization was verified using three raters. Results: 14 categories of proximal determinants were identified. Of these, environmental determinants were the largest contributor to falls (74%). Participants with poor mobility and executive function were more likely to fall while performing activities of daily living, specifically while trying to stand and bending over. However, participants with poor mobility also had lower likelihood of falling to environmental hazards and dual-tasking cognition. In contrast, high-functioning older adults with naturally fast movement speed tend to fall to environmental factors while engaging in complex motor activities. Conclusions: Our results suggest there may be two populations of fallers, the healthy and the disabled, each with their own set of distinct risk factors and triggers. Cognitively functional older adults who choose to engage in vigorous activities in hazardous environments may increase their chances of falling to dual-task cognition. Community fall prevention efforts may benefit from examining the needs of specific subpopulations.

